# Erythema nodosum

**DOI:** 10.1007/s00393-024-01529-4

**Published:** 2024-06-17

**Authors:** Viktoria Weber, Konstantin Weimann, Isabel Kolm, Barbara Meier-Schiesser

**Affiliations:** 1 Dermatologische Klinik, Universitätsspital Zürich, Rämistr. 100, 8901 Zürich, Schweiz; 2 Pathologisches Institut, Luzerner Kantonsspital, Spitalstr., 6000 Luzern 16, Schweiz

**Keywords:** Septale Pannikulitis, Sarkoidose, Streptokokken, Entzündliche Knoten, Arzneimittelreaktion, Septal panniculitis, Sarcoidosis, Streptococci, Inflammatory nodules, Drug reaction

## Abstract

Das Erythema nodosum (EN) ist die am häufigsten auftretende Form einer akuten Pannikulitis. Es wird charakterisiert durch schmerzhafte, rote bis livide, erhabene Knötchen oder Beulen, die typischerweise symmetrisch im Bereich der Schienbeine auftreten. Häufig handelt es sich beim EN um eine Reaktion des Immunsystems auf Infektionen, entzündliche Erkrankungen oder Medikamente. In etwa der Hälfte der Fälle ist kein Auslöser zu eruieren. Nach Behandlung der zugrunde liegenden Ursache ist das EN in der Regel selbstlimitierend.

## Abstract (en)

Erythema nodosum (EN) is the most frequently occurring form of acute panniculitis. It is characterized by painful red to livid raised nodules or bumps that typically occur symmetrically in the shin area. The cause of EN is often a reaction of the immune system to various triggers including infections, inflammatory diseases or medications. In approximately half of the cases no trigger can be identified. After treatment of the underlying pathology EN is typically self-limiting.

## Lernziele

Nach der Lektüre dieses Beitrags ...

- wissen Sie, dass das Erythema nodosum die häufigste Form einer septalen Pannikulitis ist,
- kennen Sie die häufigsten Auslöser des Erythema nodosum,
- sind Sie in der Lage, die verschiedenen diagnostischen Maßnahmen richtig einzusetzen,
- ist Ihnen bekannt, im Rahmen welcher Syndrome das Erythema nodosum auftreten kann.

## Einleitung

Das Erythema nodosum (EN) ist die häufigste Form einer **akuten Fettgewebsentzündung**

akuten Fettgewebsentzündung

(Pannikulitis) und ist durch schmerzhafte, subkutane, erythematöse Knoten im Bereich der Unterschenkel gekennzeichnet. Die Erkrankung tritt beim weiblichen Geschlecht 3‑ bis 6‑mal häufiger auf als beim männlichen, am häufigsten betroffen sind junge Frauen [1]. Das EN tritt bei **systemischen Erkrankungen**

systemischen Erkrankungen

auf, kann aber häufig nur als **idiopathisch**

idiopathisch

klassifiziert werden. Aufgrund der Vielzahl an möglichen Auslösern ist es entscheidend, anhand einer genauen Anamnese jene Patienten zu identifizieren, bei denen aufgrund des Verdachts auf ein EN im Kontext einer relevanten Systemerkrankung eine umfassende weitere Diagnostik erforderlich ist.

## Kurzkasuistik

Bei Ihnen stellt sich eine 27-jährige Frau vor, diese klagt neben allgemeinem Krankheitsgefühl und Fieber über stark schmerzhafte Beulen an den Unterschenkeln. Die Beulen seien so stark druckempfindlich, dass sie nicht einmal die Berührung durch die Bettdecke tolerieren könne. Seit einigen Tagen habe sie zudem auch Schwellungen und Schmerzen an beiden Sprunggelenken bemerkt. Sie nehme bis auf ein orales Kontrazeptivum keine Medikamente ein, Vorerkrankungen oder Allergien seien nicht bekannt. In der klinischen Untersuchung der febrilen Patientin (T: 38,7 °C) fallen Ihnen im Bereich der Schienbeine erythematöse, bis zu 5 cm große, stark druckdolente Knoten und Beulen auf, eine lokale Lymphadenopathie besteht nicht. Das obere Sprunggelenk (OSG) ist beidseits leicht geschwollen, gerötet, der Bewegungsumfang schmerzbedingt eingeschränkt. Sie führen eine Blutuntersuchung durch und fertigen ein Röntgenbild der Lunge sowie einen Ultraschall des OSG an. Laborchemisch zeigten sich eine Leukozytose, eine Erhöhung der BSG (Blutkörperchensenkungsgeschwindigkeit) sowie des CRP (C-reaktives Protein). Radiologisch zeigt sich eine bihiläre Lymphadenopathie, die Sonographie des OSG zeigt keine spezifischen Veränderungen, insbesondere keinen Gelenkerguss. Eine ergänzende magnetresonanztomographische Untersuchung zeigt entzündliche Veränderungen im subkutanen Fettgewebe und lediglich minimale Flüssigkeitsansammlungen im Gelenk, jedoch ohne floride Synovialitis.

Welche Verdachtsdiagnose haben Sie? Wie würden Sie weiter vorgehen?

## Epidemiologie

Obwohl das EN grundsätzlich mit etwa 1–5/100.000 Fällen eine seltene Erkrankung ist, ist es die am häufigsten auftretende **Pannikulitis**

Pannikulitis

[2]. Bei Erwachsenen liegt die **Prävalenz**

Prävalenz

von Frauen im Vergleich zu Männern bei 3–6:1 [1, 3], dabei sind insbesondere junge Frauen zwischen dem 20. und 30. Lebensjahr betroffen [2].

### Merke

Das Erythema nodosum tritt gehäuft bei jungen Frauen auf.

## Pathogenese

Beim EN handelt es sich um ein unspezifisches Reaktionsmuster der Haut auf eine heterogene Gruppe an Antigenen. Obwohl eine **Typ-IV-Hypersensitivitätsreaktion**

Typ-IV-Hypersensitivitätsreaktion

vermutet wird, ist der genaue Pathomechanismus bis heute nicht bekannt [2]. Es wird angenommen, dass sich Immunkomplexe in den Gefäßen der Septen des subkutanen Fettgewebes ablagern und dadurch zu einer neutrophilen Pannikulitis führen [4].

## Ätiologie

In etwa der Hälfte der Fälle kann kein Auslöser identifiziert werden [5, 6]. In den übrigen Fällen sind die Auslöser meist **Infektionen**

Infektionen,

vorrangig der oberen Atemwege durch Streptokokken, entzündliche Grunderkrankungen wie die Sarkoidose, Morbus Behçet, Spondyloarthritiden oder chronisch entzündliche Darmerkrankungen, neutrophile Dermatosen, Medikamente oder Schwangerschaft [7].

### Infektionen

Der mit Abstand häufigste Auslöser weltweit (ca. 30 %) des EN ist eine Infektion mit β‑hämolysierenden Streptokokken der Lancefield-Gruppe A. Dabei treten die EN-typischen Hautveränderungen ca. 2 bis 3 Wochen nach einer durchgemachten **Streptokokkenpharyngitis**

Streptokokkenpharyngitis

auf. Sollten Patienten anamnestisch Symptome einer oberen Atemwegsinfektion schildern, empfiehlt sich daher zusätzlich zur Basisdiagnostik die Durchführung eines **Rachenabstriches**

Rachenabstriches

[2]. Auch bakterielle oder virale Infektionen der unteren Atemwege oder gastrointestinale Infektionen können zum Auftreten eines EN führen (Tab. 1; [7]).

**Tab. 1:** Ursachen des Erythema nodosum [8]

*Idiopathisch (ca. 50* *%)*	
*Infektionen*	Infekte der oberen Atemwege – Streptokokken (ca. 30 %) – Respiratorische Viren (z. B. SARS-CoV-2)
	Gastrointestinale Infektionen Yersinien > Salmonellen > Campylobacter
	Pneumonien Mykoplasmen > Chlamydien > Tuberkulose
*Entzündliche Erkrankungen*	Sarkoidose (ca. 25 %)
	Chronisch entzündliche Darmerkrankungen Morbus Crohn > Colitis ulcerosa
	Neutrophile Dermatosen – Morbus Behçet (ca. 50 %) – Sweet Syndrome
*Medikamente*	Orale Kontrazeptiva, Östrogene (ca. 10 %)
	Antibiotika (Penicilline, Sulfonamide)
	TNF-Inhibitoren (Infliximab, Adalimumab)
	Checkpointinhibitoren (Nivolumab)
	BRAF-Inhibitoren (Vemurafenib)
*Sonstiges*	Schwangerschaft (5 %)
	Malignome

*TNF* Tumornekrosefaktor, *BRAF* V-Raf Murine Sarcoma Viral Oncogene Homolog B

### Entzündliche Erkrankungen

#### Sarkoidose

Bei der Sarkoidose handelt es sich um die häufigste **granulomatöse Erkrankung**

granulomatöse Erkrankung

in Nordeuropa. Diese Autoimmun- und Multisystemerkrankung ist der zweithäufigste bekannte Auslöser eines EN weltweit [9]. Etwa ein Viertel aller Sarkoidosepatienten weisen eine Hautbeteiligung auf [1]. Insbesondere ist hier das **Löfgren-Syndrom**

Löfgren-Syndrom

zu nennen, eine Form der akuten Sarkoidose. Klassischerweise wird dies durch die Trias aus Erythema nodosum, Arthritis der Sprunggelenke und bihiläre Lymphadenopathie, häufig in Kombination mit Fieber, definiert. Allerdings gilt es zu beachten, dass bei manchen Patienten weder arthrosonographisch noch magnetresonanztomographisch Zeichen einer Arthritis bzw. ein Gelenkerguss festzustellen sind [10]. Tatsächlich handelt es sich jedoch häufig um eine diffuse, perimalleoläre und nicht zwingend nodöse **periartikuläre Pannikulitis**

periartikuläre Pannikulitis,

ein sog. kontusiformes („prellungsähnliches“) EN [11]. Diese Veränderungen gehen meist dem typischen Erscheinungsbild des EN im Rahmen eines Löfgren-Syndroms voraus (Abb. 3a). Des Weiteren ist nicht in allen Fällen eine typische bihiläre Lymphadenopathie in der Röntgenübersichtsaufnahme (Abb. 3b) detektierbar, sondern möglicherweise sind entsprechende Befunde hilär und mediastinal erst im Computertomogramm zu sehen.

**Abb. 1:**
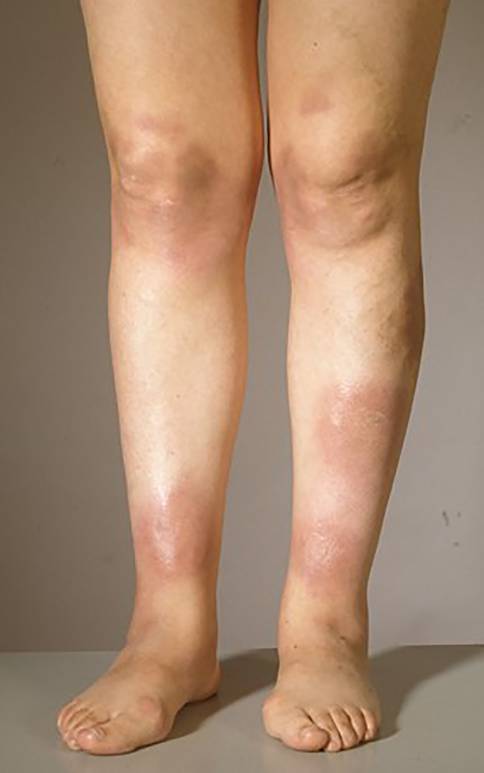
Klinisches Bild eines Erythema nodosum bei einer 37-jährigen Patientin. Multiple großflächige rote bis livide flache Knoten an den Unterschenkeln beidseits

**Abb. 2:**
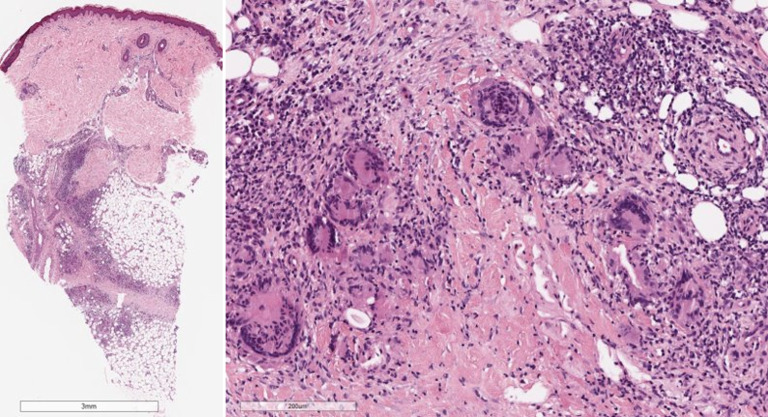
Histologisches Bild eines Erythema nodosum mit septaler Pannikulitis (Hämatoxylin-Eosin-Färbung): Das entzündliche Infiltrat ist vorwiegend auf die verdickten und fibrotischen Septen der Subkutis beschränkt. Das entzündliche Infiltrat ist überwiegend lymphozytär mit einer Beimischung von eosinophilen Granulozyten, Plasmazellen und vielen vielkernigen Riesenzellen. Die Gefäße sind unauffällig

**Abb. 3:**
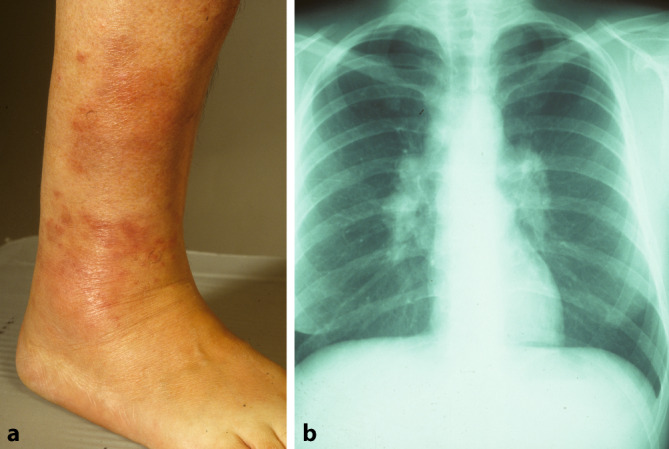
Löfgren-Syndrom bei einer 63-jährigen Patientin. **a** Rötliche bis livide Makulae im Bereich des rechten Sprunggelenks (Erythema contusiformis). **b** Röntgenaufnahme des Thorax mit bihilärer Lymphadenopathie

Das Löfgren-Syndrom ist eine spontan remittierende Erkrankung ohne Tendenz zur Rekurrenz. Wichtig hierbei zu beachten ist jedoch, dass die Kombination aus EN und vergrößerten hilären Lymphknoten auch im Rahmen eines Lymphoms oder einer Tuberkulose auftreten kann [4].

##### Merke

Das erste Symptom eines Löfgren-Syndroms ist häufig ein perimalleoläres, kontusiformes EN.

#### Chronisch entzündliche Darmerkrankungen (CED)

Hautmanifestationen im Rahmen entzündlicher Darmerkrankungen wie **Morbus Crohn**

Morbus Crohn

(MC) und **Colitis ulcerosa**

Colitis ulcerosa

(CU) sind keine Seltenheit und können mitunter der Erstmanifestation der gastrointestinalen Symptomatik vorausgehen. Diese sind vielfältig und umfassen unter anderem das das EN, Pyoderma gangraenosum, aphthöse Ulzerationen an der Mundschleimhaut, furunkuloide Granulome bis hin zur Alopecia areata [12]. Umso wichtiger ist deren korrekte Einordnung. Das EN stellt hierbei die häufigste Hautmanifestation dar und tritt bei MC in 4–15 % der Fälle und bei CU in 3–10 % auf [1]. Interessanterweise scheint das Auftreten eines EN mit der **Erkrankungsaktivität**

Erkrankungsaktivität

zu korrelieren [13].

##### Merke

Das Auftreten des EN im Rahmen einer CED korreliert mit der Krankheitsaktivität.

### Neutrophile Dermatosen

#### Morbus Behçet

Der Morbus Behçet ist eine chronisch rezidivierende **Systemvaskulitis**

Systemvaskulitis

mit bis dato unklarer Ätiologie. Gekennzeichnet ist die Erkrankung durch rezidivierende bipolare Aphten (enoral und genital), Arthritiden, Augenbeteiligung (80 %) sowie eine Hautbeteiligung, die sich in etwa 40 % der Fälle in Form eines EN äußert [3]. In anderen Fällen kann sich der Morbus Behçet kutan auch im Sinne von Papulopusteln manifestieren (ca. 50 %) [14].

##### Merke

In etwa 40 % der Fälle liegt bei einem Morbus Behçet ein Erythema nodosum vor.

#### Sweet-Syndrome

Das Sweet-Syndrom ist eine akute **febrile neutrophile Dermatose**

febrile neutrophile Dermatose,

deren Auftreten mit chronisch entzündlichen Darmerkrankungen, Schwangerschaft, Paraneoplasien (AML [akute myeloische Leukämie]) oder Medikamenteneinnahme (G-CSF [„granulocyte-colony stimulating factor“]) assoziiert ist [15]. Im Zusammenhang mit dem Sweet-Syndrom kann es auch zur Ausbildung einer **septalen Pannikulitis**

septalen Pannikulitis,

eines EN, kommen. Das gleichzeitige Auftreten beider Erkrankungen kann sowohl im Rahmen zugrunde liegender Neoplasien [16], Grunderkrankungen wie Sarkoidose [17] und MC [18] oder Streptokokkeninfektionen [17, 19] vorkommen als auch als Teil des VEXAS(„vacuoles, E1 enzyme, X‑linked, autoinflammatory, somatic“)-Syndroms. Letzteres ist eine autoinflammatorische Erkrankung von hoher Morbidität und Mortalität, die durch somatische Mutationen im *UBA1*-Gen verursacht wird und typischerweise mit Fieber, neutrophilen Dermatosen, Vaskulitis und hämatologischen Anomalien wie Myelodysplasie einhergeht [20].

Im Gegensatz dazu stellt das **subkutane Sweet-Syndrom**

subkutane Sweet-Syndrom,

eine lobuläre Pannikulitis mit neutrophilem Infiltrat [21], eine Differenzialdiagnose des EN dar.

#### Medikamente

Etwa 3–10 % der Fälle des EN sind auf eine **Überempfindlichkeitsreaktion**

Überempfindlichkeitsreaktion

auf verschiedene Medikamente zurückzuführen. Hierbei sind besonders **Antibiotika**

Antibiotika

wie Sulfonamide und Amoxicillin, aber auch orale **Kontrazeptiva**

Kontrazeptiva

zu nennen [2]. Hierbei sind besonders häufig orale östrogenhaltige Kontrazeptiva die Auslöser, gefolgt von Antibiotika, meist Sulfonamide und Amoxicillin. Ein EN kann jedoch auch durch TNF(Tumornekrosefaktor)-α-Inhibitoren, neuere Immuntherapien oder zielgerichtete Therapien von Tumoren bedingt werden ([2]; s. Tab. 1).

### Sonstige Auslöser

#### Schwangerschaft

Aufgrund der hohen Inzidenz von 5 % eines EN in der Schwangerschaft oder bei Einnahme von östrogenhaltigen Medikamenten stehen auch **weibliche Sexualhormone**

weibliche Sexualhormone

im Verdacht, einen weiteren Auslöser des EN darzustellen [2]. Unterstützt wird diese These durch die sinkende Inzidenz ab 1980, als niedriger dosierte, östrogenhaltige Kontrazeptiva auf den Markt kamen [22].

#### Malignome

In seltenen Fällen ist ein paraneoplastisch auftretendes EN **Erstmanifestation**

Erstmanifestation

eines zugrunde liegenden Malignoms. Vorrangig sind hier Hodgkin- und Non-Hodgkin-Lymphome zu nennen, aber auch im Rahmen von Leukämien und soliden Tumoren kann es zum Auftreten eines EN kommen [1].

##### Merke

Das EN kann als kutane Erstmanifestation eines Lymphoms auftreten.

## Klinik

Das Erythema nodosum tritt typischerweise akut auf und ist bei einem Drittel der Patienten vergesellschaftet mit einer vorangehenden Allgemeinzustandsverschlechterung, Fieber und Gelenkschmerzen, die typischerweise im Bereich der Sprunggelenke auftreten [6, 23]. Im Bereich der Haut finden sich erythematöse oder livide runde, unscharf begrenzte, stark schmerzhafte, nicht ulzerierende Knoten oder Plaques von bis zu 5 cm Durchmesser [24]. Das EN weist zumeist eine **symmetrische Verteilung**

symmetrische Verteilung

bilateral an den Streckseiten der Unterschenkel auf (Abb. 1), seltener sind die typischen Hautveränderungen auch an den Oberschenkeln, dem Gesäß oder den Streckseiten der Arme zu finden [25]. In einem Zeitraum von 2 bis 8 Wochen zeigt sich nach einer Abflachung der Läsionen (weniger nodöser Aspekt) eine charakteristische Farbveränderung: von anfänglich rot oder livide zu bräunlich und schließlich grünlich-gelblich, bevor das Erythema nodosum schließlich narbenfrei abheilt. Aufgrund der farblichen Veränderungen der Läsionen ähnlich einem Hämatom werden diese auch als **Erythema contusiformis**

Erythema contusiformis

(„prellungsähnliche Rötung“) bezeichnet. Nicht selten kommt es zu Rezidiven [8].

### Merke

Aufgrund der charakteristischen Farbveränderung der Läsionen im Verlauf wird das Erythema nodosum auch als Erythema contusiformis bezeichnet.

## Histopathologie

Die Histologie wird charakterisiert durch eine septale Pannikulitis ohne begleitende Vaskulitis. Die Zusammensetzung des entzündlichen Infiltrates ändert sich im Verlauf: Im **Initialstadium**

Initialstadium

zeigt sich im Korium ein schütteres perivaskuläres lymphozytäres Infiltrat, im Bereich des Fettgewebes zeigen sich septal und paraseptal Infiltrate neutrophiler Granulozyten mit einer Verbreiterung der subkutanen Septen durch das Ödem. Das **Vollbild**

Vollbild

ist gekennzeichnet durch perivaskuläre und im Fettgewebe überwiegend septale histiozytenreiche Infiltrate mit Beimengung von Riesenzellen und neutrophilen Granulozyten. In der Subkutis finden sich durch fibrotischen Umbau verdickte Septen. Charakteristisch sind die sog. **Miescher-Radiärknötchen**

Miescher-Radiärknötchen,

hierbei handelt es sich um paraseptale Granulome mit mehrkernigen histiozytären Riesenzellen (Abb. 2; [26]).

### Merke

Das Erythema nodosum ist eine septale Entzündung des subkutanen Fettgewebes ohne begleitende Vaskulitis. Spezifisch ist der Nachweis von Miescher-Radiärknötchen.

## Differenzialdiagnosen

Differenzialdiagnostisch kommen verschiedene weitere Pannikulitiden in Betracht, die ähnliche Veränderungen hervorrufen können. Hervorzuheben ist hier insbesondere das **Erythema induratum Bazin**

Erythema induratum Bazin,

das klinisch sich ebenfalls vorwiegend an den Unterschenkeln manifestiert (allerdings dorsal im Vergleich zum EN). Auch eine pankreatische Pannikulitis kann sich an den Unterschenkeln manifestieren und sollte bei Verdacht oder Persistenz der Läsionen mittels laborchemischer Untersuchungen (s. Abschnitt „Diagnostik“) ausgeschlossen werden. Des Weiteren sollten **infektiöse Quellen**

infektiöse Quellen

bei der Anamnese genau erfragt werden, da bakterielle, mykobakterielle oder Pilzinfektionen ebenfalls zu subkutanen Entzündungen führen können [27, 28]. Bei Verdacht sollte bei der Durchführung einer Biopsie ein Teil des Gewebes mikrobiologisch untersucht werden. Eine Liste genannter und weiterer möglicher Differenzialdiagnosen zeigt Tab. 2.

**Tab. 2:** Differenzialdiagnosen des Erythema nodosum. (Adaptiert nach [27, 28])

*Infektionen*	
Infektiöse Pannikulitis durch (Myko‑)Bakterien oder Pilze	Klinisch: ulzerierende Knoten ohne spezifische Lokalisation, Narbenbildung, systemische Infektzeichen
	Histologisch: septale und lobuläre neutrophilenreiche Infiltrate mit Abszessbildung, Nachweis von Erregern in den Spezialfärbungen
Erythema induratum Bazin (Tuberkulose)	Klinisch: ulzerierende Knoten im Bereich der Waden, Narbenbildung
	Histologisch: subkutane, vorwiegend lobuläre Infiltrate mit Vaskulitis mittelkalibriger Arterien
*Entzündliche Erkrankungen*	
Lupus-Pannikulitis	Klinisch: ulzerierende Knoten im Bereich der Oberarme und des Gesichts, Narbenbildung
	Histologisch: lobuläre Pannikulitis, kleine perlschnurartig um Adipozyten angeordnete Lymphozyten, keine Vaskulitis
Kutane Polyarteriitis nodosa	Klinisch: ulzerierende Knoten im Bereich der unteren Extremität, Livedo racemosa, Nekrosen
	Histologie: leukozytoklastische Vaskulitis arterieller Gefäße am Übergang zur Subkutis, keine genuine Pannikulitis
*Systemerkrankungen*	
Pankreatische Pannikulitis	Klinisch: ulzerierende Knoten, häufig gelenknah, Narbenbildung, gürtelförmige Oberbauchschmerzen
	Histologisch: lobuläre Pannikulitis, Geisterzellen und verseifte Fettläppchen
Alpha-1-Antitrypsin-Mangel-Pannikulitis	Klinisch: zu Beginn erysipelartig, im Verlauf ulzerierende Knoten, vorwiegend an Stamm, Schultern und Hüften
	Histologisch: lobuläre Pannikulitis ohne Vaskulitis, Cluster normaler Adipozyten direkt neben Nekrosearealen
*Sonstiges*	
Kältepannikulitis	Klinisch: schmerzlose Knoten, häufig Kinder, kälteexponierte Areale – Kinn, Wangen, spontane Abheilung
	Histologie: lobuläre Pannikulitis ohne Vaskulitis

## Diagnostik

In der Regel handelt es sich beim EN um eine **Blickdiagnose**

Blickdiagnose.

Aufgrund der Heterogenität der möglichen Auslöser des EN ist es jedoch wichtig, anhand einer genauen Anamnese sowie körperlichen Untersuchung die Patienten zu identifizieren, bei denen aufgrund des Verdachts auf das Vorliegen einer relevanten Systemerkrankung eine weiterführende Diagnostik angezeigt ist. Im Rahmen der **Anamnese**

Anamnese

empfiehlt es sich, die Symptomatik der wichtigsten Auslöser systematisch abzufragen und nur bei Auffälligkeiten weiterführende **laborchemische Diagnostik**

laborchemische Diagnostik

durchzuführen. Eine einheitliche Empfehlung zur Bestimmung der laborchemischen Parameter existiert nicht. Basierend auf den zugrunde liegenden Ursachen und der entsprechenden Literatur, empfiehlt sich jedoch initial die Untersuchung des Differenzialblutbildes, des C‑reaktiven Proteins, des Antistreptolysintiters (bei entsprechender Symptomatik) und des Beta-HCG (humanes Choriongonadotropin) (bei Frauen im gebärfähigen Alter) [27]. In der **körperlichen Untersuchung**

körperlichen Untersuchung

sollten neben dem Hautbefund noch eine enorale Inspektion sowie die Untersuchung der Lymphknotenstationen und der Gelenke erfolgen. Eine **Hautbiopsie**

Hautbiopsie

ist nur bei atypischen Läsionen oder Persistenz trotz Therapie nötig, in diesen Fällen empfiehlt sich die Durchführung einer tiefen Spindelbiopsie. Sollten sämtliche Untersuchungsergebnisse unauffällig ausfallen, handelt es sich aller Wahrscheinlichkeit nach um ein idiopathisches EN. Von weiterführender Diagnostik ist in diesen Fällen abzusehen [2, 4, 6, 23].

### Merke

Eine Hautbiopsie ist in der Regel nicht notwendig.

## Therapie

In der Mehrzahl der Fälle ist das EN selbstlimitierend und eine rein **symptomatische Therapie**

symptomatische Therapie

ausreichend. Falls diese zu eruieren ist, sollte die ursächliche Grunderkrankung behandelt oder das auslösende Medikament abgesetzt werden. **Erstlinientherapie**

Erstlinientherapie

sind Hochlagerung der Beine und Kompression sowie eine analgetische und antiphlogistische Therapie mit **nichtsteroidalen Antirheumatika**

nichtsteroidalen Antirheumatika

(NSAR), z. B. Ibuprofen 400–800 mg bis zu 4-mal täglich. (Maximaldosis 3200 mg/Tag). Häufig werden zusätzlich **topische Glukokortikoide**

topische Glukokortikoide

der Klasse IV in Kombination mit **Dimethylsulfoxid**

Dimethylsulfoxid

(DMSO) 1:1 eingesetzt, um die Eindringtiefe in das Gewebe zu erhöhen. Ebenfalls eine hohe Wirksamkeit weist die perorale Gabe von **Kaliumjodid**

Kaliumjodid

3‑mal täglich 300 mg für die Dauer von 1 bis 2 Wochen auf [29]. Bei Patienten, die auf NSAR und Kaliumjodid nicht ansprechen oder primär eine Konstellation mit starken Schmerzen und/oder substanziell erhöhten Entzündungsparametern aufweisen, können **systemische Glukokortikoide**

systemische Glukokortikoide

zum Einsatz kommen, worunter sich in der Regel ein schnelles Krankheitsansprechen zeigt (Dosen variieren zwischen 0,25 und 1 mg/kgKG [Körpergewicht] [27, 30, 31], langsames Ausschleichen in 10- bis 20 mg- und ab 20 mg in 5 mg-Schritten). Zum Einsatz von immunmodulatorischen und immunsuppressiven Therapien wie Colchicin, Hydroxychloroquin oder Dapson existieren nur Einzelfallberichte. Diese sind angezeigt im Falle therapierefraktärer oder chronisch rezidivierender Verläufe [32].

## Fazit für die Praxis

- Bei dem Erythema nodosum (EN) handelt es sich um die häufigste Form einer septalen Pannikulitis, betroffen sind überwiegend junge Frauen.
- Mögliche Auslöser sind Infektionen, Medikamente, Sarkoidose und andere Systemerkrankungen sowie Schwangerschaft.
- In den meisten Fällen lässt sich kein expliziter Auslöser eruieren.
- Das EN ist eine Blickdiagnose, aufgrund der charakteristischen Farbveränderung der Läsionen im Verlauf kann es mit einem Hämatom verwechselt werden.
- Diagnostisch wichtig sind eine gründliche Anamnese und körperliche Untersuchung; eine Biopsie ist nur in Ausnahmefällen erforderlich.
- Ein wichtiges differenzialdiagnostisches Kriterium zur Abgrenzung des EN von anderen Pannikulitiden ist dessen fehlende Ulzeration.
- Die Erstlinientherapie des EN besteht aus nichtsteroidalen Antirheumatika (NSAR), Kompression/Hochlagerung der betroffenen Extremität und topischen Glukokortikoiden in Kombination mit Dimethylsulfoxid (DMSO).
- Bei Vorliegen von entzündlichen Systemerkrankungen sind häufig systemische Glukokortikoide notwendig.

## CME-Fragebogen

Wo befindet sich histopathologisch primär die Entzündung beim Erythema nodosum?

Haarfollikel

Synovia

Epidermis

Talgdrüsen

Subkutanes Fett

Bei einer Patientin mit Erythema nodosum (EN) steht eine mögliche Sarkoidose im Raum. Was ist der nächste diagnostische Schritt?

CT (Computertomographie) des Thorax/Abdomens

Serologie mit ANA(antinukleäre Antikörper)- und ANCA(antineutrophile zytoplasmatische Antikörper)-Bestimmung

Röntgenaufnahme des Thorax

Arthrosonographie der Hände

Quantiferon-Test

Welcher histologische Befund ist besonders typisch für eine Sarkoidose?

Verkäsende Granulome

Nekrotisierende Vaskulitis

Miescher-Radiärknötchen

Granulomatöse Entzündung mit Touton-Riesenzellen

Fibrilläre Fibrosierung

Bei einem Hautbefund wird ein Erythema nodosum (EN) vermutet. Welcher klinische Befund passt am ehesten zum EN?

Ulzerationen im Bereich der Knöchel

Asymmetrische Verteilung im Bereich der Extremitäten

Starker Juckreiz der Läsionen

Klinische Ähnlichkeit zu einem Hämatom

Läsionen bestehen über mehrere Jahre.

Das Löfgren-Syndrom ist eine akute Form der Sarkoidose. Was gehöht neben Erythema nodosum (EN) und Arthritis noch dazu?

Hyperkalzämie

Hyperkalziurie

Perikarditis

Periphere Polyneuropathie

Hiläre Lymphadenopathie

Was ist der häufigste bekannte Auslöser eines Erythema nodosum (EN)?

Viraler gastrointestinaler Infekt

Streptokokkeninfektion

Sonnenexposition

Vorangegangenes Trauma

Tumorleiden (Paraneoplasien)

Bei einer Patientin wird ein Erythema nodosum (EN) vermutet. Die Diagnose ist unsicher und soll unbedingt gesichert werden. Welche Diagnostik bietet sich an?

Sonographie

Szintigraphie

Lymphknotenbiopsie

Feinnadelbiopsie

Spindelbiopsie

Welche Therapie wird bei schweren Verläufen des Erythema nodosum (EN) am ehesten eingesetzt?

Rituximab

Kortikosteroide

TNF(Tumornekrosefaktor)-Inhibitoren

JAK(Januskinase)-Inhibitoren

Cyclophosphamid

Eine junge Frau stellt sich mit Erythema nodosum (EN) vor. Welche Therapie sollte initial am ehesten durchgeführt werden?

Dapson

Kolchizin

Ibuprofen

Abatacept

Azathioprin

Ein 1‑jähriger Junge stellt sich mit schmerzlosen Knoten im Bereich der Wangen vor. Was ist die wahrscheinlichste Ursache?

Kutanes T‑Zell-Lymphom

Kälteexposition

Sarkoidose

Morbus Behçet

Sweet-Syndrom
